# Molecular insights into NLRP3 inflammasome and miRNA modulation in oral cancer

**DOI:** 10.3389/fphar.2025.1713259

**Published:** 2026-01-05

**Authors:** Deborah Mannino, Morena D’Ariano, Ivana Bello, Irene Paterniti, Giovanna Casili, Elisabetta Panza

**Affiliations:** 1 Research Operative Unit of Neuropharmacology and Translational Neurosciences, Oasi Research Institute, Troina, Italy; 2 Department of Pharmacy, University of Naples Federico II, Naples, Italy; 3 Department of Chemical, Biological, Pharmaceutical and Environmental Sciences, University of Messina, Messina, Italy

**Keywords:** cancer, inflammation, microRNAs, NLRP3 inflammasome, oral squamous cell carcinoma

## Abstract

The NLRP3 inflammasome, a cytosolic multiprotein complex composed of NLRP3, ASC, and caspase-1, orchestrates the maturation of interleukin-1β (IL-1β) and interleukin-18 (IL-18) and the induction of pyroptosis, acting as a central mediator of innate immunity. Although physiologically protective, aberrant NLRP3 activation has been increasingly implicated in tumorigenesis. In oral squamous cell carcinoma (OSCC), current evidence points to a predominantly pro-tumorigenic role, with elevated NLRP3 expression correlating with tumor progression, lymph node metastasis, advanced pathological stage, and reduced survival. Functional studies demonstrate that genetic silencing or pharmacological inhibition of NLRP3 enhances apoptosis and reduces tumor burden. An additional regulatory layer is provided by microRNAs (miRNAs), which fine-tune NLRP3 expression at the post-transcriptional level. Since the identification of miR-223-3p as the first miRNA to directly target NLRP3, several miRNAs, including miR-22-3p, miR-7-5p, and miR-30e-5p, have been shown to suppress NLRP3 activity in various pathological settings, including oral squamous cell carcinoma, where miR-22-3p downregulates NLRP3, inhibiting its proliferation, migration, and invasion. Therefore, the NLRP3 inflammasome represents a key player in cancer development, and its regulation by miRNAs highlights its importance and clinical potential. This review summarizes mechanistic and clinical knowledge on the biology of NLRP3, highlights its dual role in cancer hallmarks, and discusses the therapeutic promise of targeting the NLRP3-miRNA axis in the management of oral cancer.

## 1 Introduction

The NLRP3 inflammasome, a cytosolic multiprotein complex composed of the sensor molecule NLRP3, the adaptor caspase recruitment domain (ASC), and caspase-1, serves as a pivotal mediator of innate immunity through maturation and secretion of pro-inflammatory cytokines such as interleukin-1β (IL-1β) and interleukin-18 (IL-18), and the induction of pyroptosis. While physiologically critical for host defense, aberrant activation of the NOD-like-, leucine-rich repeat (LRR)-, and pyrin domain (PYD)-containing protein (NLRP3) inflammasome has been implicated in multiple pathologies, including tumorigenesis (Scuderi et al., 2021). Recent investigations have revealed a dual role of NLRP3 in cancer, acting variably as a tumor promoter or suppressor depending on cellular context and tumor type.

In the context of oral squamous cell carcinoma (OSCC), accumulating evidence indicates that NLRP3 predominantly exerts a pro-tumorigenic function. Elevated expression of NLRP3 correlates with enhanced tumor growth, lymph node metastasis, and IL-1β expression; conversely, NLRP3 knockdown impairs proliferation, invasion, and metastatic capacity both *in vitro* and *in vivo* (Wang et al., 2018). These observations point to NLRP3 as a key modulator of the inflammatory tumor microenvironment, which is a hallmark of OSCC progression. Inhibiting NLRP3 using the small molecule BAY-117082 effectively reduces OSCC cell viability, suppresses inflammasome activation (including ASC, caspase-1, IL-1β, and IL-18), promotes pro-apoptotic markers (e.g., Bax, Bad, p53) while downregulating Bcl-2, and significantly decreases tumor burden in xenograft models (Scuderi et al., 2021). Clinically, increased NLRP3 expression together with elevated serum IL-1β levels have been associated with disease severity in potentially malignant oral disorders and OSCC, supporting its value as a diagnostic and prognostic biomarker (Jain et al., 2024). NLRP3 levels and downstream effectors, like caspase-1 and IL-1β, can be measured as direct biomarkers and surrogate biomarkers by immunohistochemistry, qRT-PCR in tumor tissues of biofluids. Cut-off of these biomarkers depends on cancer type. For example, prostate cancer studies show significantly higher NLRP3 expression related with tumor cells proliferation and invasion. The evaluation of cut-offs should be established comparing high *versus* low expression patient groups and monitoring important parameters such us survival and treatment response during the follow-up. The cell viability and proliferation could be evaluated by measuring NLRP3 activation via IL-1β and NF-kB pathways (Sun et al., 2025). Further mechanistic insights reveal that the NLRP3 inflammasome may also contribute to chemoresistance; genetic ablation of NLRP3 enhances the antitumor efficacy of 5-fluorouracil (5-FU) in OSCC models, delaying tumor onset and reducing tumor burden (Pajak et al., 2017). This link between NLRP3 activation and treatment resistance provides additional rationale for exploring NLRP3-based therapeutic interventions. Collectively, these studies converge to highlight the NLRP3 inflammasome as a central driver of OSCC pathogenesis and a promising therapeutic target. For these reasons, clarifying how NLRP3 shapes OSCC initiation, progression, and response to therapy is essential to understanding its potential in future clinical strategies. This review aims to synthesize the latest advances on NLRP3 inflammasome biology, its dichotomous involvement in cancer hallmarks, and its specific contributions to OSCC onset, progression, and therapeutic resistance. Through a critical evaluation of mechanistic and translational findings, we seek to delineate the potential of NLRP3-targeted strategies within oral cancer management and to identify open questions that warrant further investigation.

## 2 The biology of NLRP3 inflammasome

**FIGURE 1:**
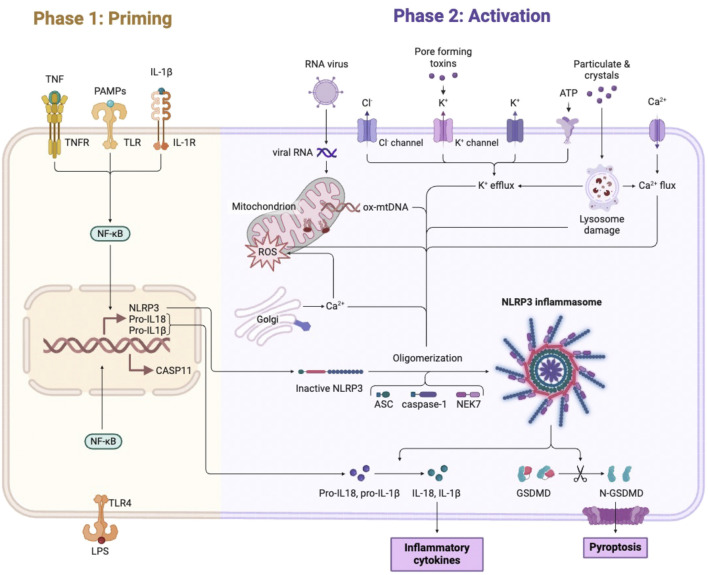
Overview of the canonical priming and activation of the NLRP3 inflammasome. Phase 1 (priming, left) is induced by TLRs, tumor necrosis factor receptors (TNFRs), which may recognize PAMPs and increase the transcriptional levels of NLRP3 heat domain-associated protein, pro-IL-1β, and pro-IL-18 cytokines via the NF-κB pathway. Phase 2 (activation, right) occurs after priming and involves the formation of the NLRP3 inflammasome from its constituent proteins (NLRP3, ASC, and caspase-1), leading to the maturation of IL-1β and IL-18. Multiple mechanisms activate the inflammasome, such as extracellular ATP, potassium efflux, altered calcium signaling, lysosomal destabilization, and mitochondrial dysfunction products such as mitochondrial DNA and ROS (Created by BioRender.com).

The inflammasome is a key component of the innate immune system response to pathogens and consists of the NOD-like receptor (NLR) family and the apoptosis-associated adaptor protein (ASC). The formation of these protein complexes leads to the activation of caspase-1 to promote pyroptosis, a proinflammatory programmed cell death that results in the release of cellular contents (Huang Y. et al., 2021). Among inflammasomes, the NLRP3 inflammasome is the most widely studied and is activated by a broad spectrum of stimuli. (Swanson et al., 2019). The activation mechanism of the NLRP3 inflammasome can occur by either a canonical or a non-canonical pathway. Canonical NLRP3 activation occurs in two phases: priming and activation. The priming phase is triggered by pattern-recognition receptors such as Toll-like receptor 4 (TLR4) or tumor necrosis factor (TNF) receptors. These signals activate NF-κB, which increases the transcription of NLRP3, pro-IL-1β, and pro-IL-18. (Bauernfeind et al., 2009). The activation phase is initiated by a wide range of pathogen- or damage-associated signals (PAMPs and DAMPs). These include extracellular ATP, LPS, ion flux, lysosomal damage, and mitochondrial dysfunction with reactive oxygen species (ROS) production. Together, these stimuli promote inflammasome assembly. (Xue et al., 2019). Both phases are triggered by microbial or sterile inflammatory stimuli, although microbial signals are different from sterile signals in terms of kinetics and magnitude (Figure 1). Non-canonical NLRP3 activation occurs mainly in response to intracellular LPS from Gram-negative bacteria. LPS enters the cytoplasm independently of TLR4 and directly activates pro-caspase-11. Caspase-11 then cleaves Gasdermin-D, inducing pyroptosis and secondarily activating NLRP3. Intracellular LPS binds to and activates pro-caspase-11, which induces the cleavage of GSDMD, leading to pyroptosis and activation of the NLRP3 inflammasome (Kayagaki et al., 2013). NLRP3 is expressed in myeloid lineage immune cells such as neutrophils, monocytes, dendritic cells, lymphocytes, and neurons (Yu and Lee, 2016). In contrast, abnormal activation is closely related to several autoinflammatory disorders, including cryopyrin-associated autoinflammatory syndrome (CAPS) (Putnam et al., 2024), diabetes, gouty arthritis, atherosclerosis (Hu et al., 2024), inflammatory bowel disease (IBD) (Chen Q. L. et al., 2021), Parkinson’s disease (PD), and Alzheimer’s disease (AD) (Lu et al., 2022). In addition, NLRP3 inflammasome plays a crucial role in cancer and metabolic diseases (Tengesdal et al., 2023). Therefore, understanding the mechanism and consequences of NLRP3 inflammasome activation in cancer is critical for developing novel therapeutic strategies. Moreover, several NLRP3 inflammasome inhibitors have been recently developed and studied in different pathologies, some of which are advancing toward clinical trials (Nelson et al., 2025; Gatlik et al., 2024). Early NLRP3 inhibitors included indirect modulators such as resveratrol, arglabine, glyburide, and β-hydroxybutyrate (BHB). However, these compounds affected multiple signaling pathways and caused significant off-target effects. To overcome these limitations, several selective NLRP3 inhibitors have been developed, including MCC950, BAY-117082, CY-09, OLT1177, tranilast, MNS, and oridonin. These molecules target NLRP3 or its oligomerization more specifically and therefore reduce off-target immunosuppression (Table 1). By directly targeting the NLRP3 protein or its complex, these inhibitors can reduce unwanted side effects and off-target immunosuppressive effects (Coll et al., 2015). Over the past decade, studies have focused on investigating there *in vitro* and *in vivo* effects on inflammatory diseases, neurodegenerative diseases, and some types of cancer (Casili et al., 2024). Among these inhibitors, the small molecule NLRP3 inhibitor MCC950 has shown promising results in various disease models, including autoinflammatory disorders, cardiovascular diseases, cancer, neurological diseases, and diabetes. MCC950 inhibits ATP hydrolysis and inflammasome formation by binding to the NACHT domain of the NLRP3 protein (Coll et al., 2019). Another inhibitor, BHB, reduces K^+^ efflux and ASC aggregation, thereby preventing NLRP3 activation and lowering IL-1β and IL-18 levels in human monocytes. BHB has shown anti-inflammatory effects in models of Alzheimer’s disease, gout, and acute kidney injury and has potential as a clinical treatment (Shippy et al., 2020). However, the clinical use of NLRP3 inhibitors still faces challenges related to pharmacokinetics and hepatotoxicity. Despite these advances, the translational development of NLRP3 inhibitors remains limited. Their clinical applicability is currently limited by suboptimal pharmacokinetic profiles, the risk of systemic immunosuppression, and hepatotoxicity, factors that limit their long-term use in oncology (Nizami et al., 2021). Furthermore, although new-generation inhibitors demonstrate greater specificity for the NLRP3 complex, they still lack tumor-selective activity, raising concerns about off-target effects in tissues with physiological inflammasome functions (Zahid et al., 2019). Importantly, only a few NLRP3-targeting compounds, such as DFV890, have entered early-stage clinical trials, and none have yet progressed to cancer studies (Gatlik et al., 2024). Therefore, the therapeutic potential of these agents in oncology remains largely unexplored in clinical settings.

**TABLE 1:** Summary table of alternative strategies in the regulation of NLRP3 inflammasome via miRNAs and possible molecular outcomes in cancer.

Type of cancer	miRNAs	Regulation mechanisms	References
OSCC Breast cancer Non-small cell lung carcinoma Hepatocellular carcinoma	miR-223-3p	• Suppression pro-inflammatory cytokines IL-1β and IL-18 • Inhibits cancer progression thought decrease of cell proliferation, increase apoptosis, inhibition migration and invasion	Zhu et al. (2022) Zhang et al. (2019), Wan et al. (2018)
Breast cancer OSCC Melanoma	miR-22-3p	• Suppression pro-inflammatory cytokines IL-1β and IL-18 • Enhancement of anti-cancer immunity • Reduction of cancer cell proliferation, invasion, and migration	Zhang et al. (2019), Wan et al. (2018) Liu et al. (2024) Gorur et al. (2021)
Breast cancer Non-small cell lung cancer Colorectal cancer Bladder cancer Glioblastoma	miR-7-5p	• Reduction NLRP3 inflammasome activation • Suppression pro-inflammatory cytokines IL-1β and IL-18 • Promotes apoptosis and inhibition of proliferation and invasiveness	Korac et al. (2021)
Non-small cell lung cancer Head and neck squamous cell carcinoma Colorectal cancer Breast cancer	miR-30e-5p	• Inhibition of NLRP3 activation and related pro-tumor inflammation • Modulation key oncogenic genes involved in tumor growth, metastasis and angiogenesis	Zhang et al. (2019), Wan et al. (2018)
Colorectal cancer Gastric cancer Lung cancer	miR-186-5p	• Reduction of NLRP3 activation and secretion pro-inflammatory cytokines	Tezcan et al. (2019)
Lung cancer Esophageal cancer	miR-495-3p	• Negatively regulation of NLRP3 complex activation • Decrease of inflammasome assembly • Decrease caspase-1 activation • Suppression pro-inflammatory cytokines IL-1β and IL-18	Tezcan et al. (2019)

## 3 Divergent function of NLRP3 inflammasome in cancer

**FIGURE 2:**
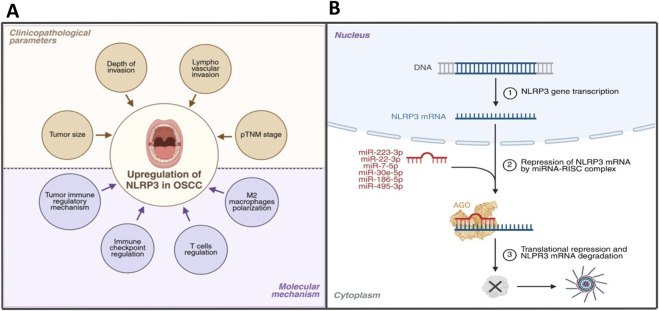
Divergent role of NLRP3 inflammasome in cancer. The role of NLRP3 inflammasome is more crucial both as oncogenic and onco-suppressor factor in a fragile balance between tumor growth and tumor restraint. NLRP3 inflammasome activates various molecular pathways involved in the management of tumor-related inflammation. The downstream effects mainly depend on the type and stage of the tumor, related to clinical-pathological features of specific cancers. (created with BioRender).

The cancer-related inflammation plays an important role in tumor escape from immunological clearance, which is an important feature in aggressiveness and malignancies (de Visser and Coussens, 2006; Mahjoor et al., 2021; Shadab et al., 2023). Lately, immunity-cancer association has been studied a lot since cancer cells can be distinguished from “normal” self-cells by their different biochemical make-up, antigenic structure, and biological behaviour (Olszanski, 2015; Pardoll, 2015) in order to evade the immune system to grow unchecked (Abbott and Ustoyev, 2019). Immunosuppression is not the only determinant of tumor progression; nonetheless, its effects are frequently mediated through chronic inflammatory pathways that critically sustain tumor development. By infiltrating the tumor microenvironment (TME), releasing cytokines, growth factors, chemokines, and proangiogenic factors, and causing genome instability and immune evasion, inflammation can increase the risk of cancer-promoting cells (Galdiero et al., 2018). One of the peculiarities of cancer, in fact, is chronic inflammation that has been recognized as the “seventh hallmark of cancer” (Mantovani, 2009). Numerous cell types, including fibroblasts, endothelial cells, macrophages, and tumor cells, produce proinflammatory cytokines during the inflammation process, which have the ability to encourage the onset, development, and metastasis of various cancers (Balahura et al., 2020). Recently, the study of tumor-associated inflammation has been enriched with new actors, in particular a complex known as inflammasomes. In this context, the NLRP3 inflammasome’s overexpression has been linked to a range of malignancies (Wang et al., 2018) and its divergent role in different tissue tumor types, such as bladder urothelial carcinoma (BLCA), breast invasive carcinoma (BRCA), colon adenocarcinoma (COAD), lung adenocarcinoma (LUAD), lung squamous cell carcinoma (LUSC), rectum adenocarcinoma (READ), kidney renal clear cell carcinoma (KIRC), and stomach adenocarcinoma (STAD) in which are both downregulated and upregulated (Wang et al., 2022), has been extensively discussed. In this context, the NLRP3 inflammasome is crucial for its divergent role, because it is involved both in cancer progression and regression (Hamarsheh and Zeiser, 2020). The NLRP3 activation influences the crosstalk between innate and adaptive immunity by modulating immune cell recruitment, cytokine secretion, and T-cell differentiation. Understanding these mechanisms is crucial in the modulation of inflammasomes for therapeutic approaches since inflammasomes can contribute to tumor growth and metastasis through chronic inflammation, but their components are key in control of tumor growth and metastasis restrain and represent novel therapeutic possible targets. These therapeutic strategies aim to modulate inflammasome activity to enhance anti-tumor immune responses and improve clinical outcomes (Singh et al., 2025). Considering the complexity of the potential role of NLRP3 inflammasome in tumorigenesis, immune regulation, and functional differences among inflammasome family members, results require deeper analysis on the relationships between NLRP3 inflammasome and specific tumors. In particular, the increased expression of NLRP3 in human Breast Cancer Associated Fibroblasts (CAFs) is a precursor to cancer progression and metastasis through secretion of IL-1β (Saponaro et al., 2021) which stimulates angiogenesis, immune evasion, epithelial-mesenchymal transition (EMT), and stemness (Saponaro et al., 2021). Moreover, the role of NLRP3 inflammasome in breast cancer may depend on the TME and the subtypes of breast cancer. For example, NLRP3 and IL-1β overexpression in tumor-associated macrophages (TAMs), was associated with survival, lymph node invasion, and metastasis in patients with HER2+ breast cancer (Weichand et al., 2017), and interestingly, in murine invasive breast cancer models, the absence of a functional NLRP3 inhibited tumor growth (Guey et al., 2020). In the context of this disease, due to its overexpression in tumor areas and its association with larger tumor size, higher histological grade, positive node and receptor status, the NLRP3 inflammasome appears to be involved in tumor aggressiveness (Saponaro et al., 2021). In lung cancer, particularly in Non-Small Cell Lung Cancer (NSCLC), TAMs in the TME play an important role because they are associated with the production of various pro-inflammatory cytokines and chemokines (IL-1β, IL-6, IL-8, IL-12, and IL-18), promoting tumor growth and metastasis (Dey Sarkar et al., 2021). Particularly, IL-1β and IL-18 in this context create a favorable environment for tumor growth and survival by increasing migration and invasion of tumor cells through the modulation of EMT, matrix metalloproteinases (MMPs) (Liu et al., 2022) and also, by inducing the expression of vascular endothelial growth factor (VEGF) that could play a role in the development of the premetastatic niche (Shi et al., 2017; Chai et al., 2020). Additionally, inflammasome activation can suppress the anti-tumor immune response by the secretion of immunosuppressive cytokines such as IL-10 and transforming growth factor beta (TGF-β), the polarization of macrophages M2 phenotype, and by inhibiting T and natural killer (NK) cells activities (Wisitpongpun et al., 2022). Therefore, NLRP3 inflammasome has a complex and dual role in NSCLC depending on the context and stage of the disease. Prostate cancer is characterized by high levels of IL-1β, IL-18, IL-6, and MIC-1/GDF-15 that are clinically relevant for this association with the risk of carcinoma and the prognosis of established cancer. NLRP3 inflammasome is a key regulator of inflammation in this cancer for enhance tumor cell growth, survival, migration, and invasion by regulating autophagy, mitochondrial metabolism, EMT (Xu Z. et al., 2021), angiogenesis, immune evasion, and metastasis (Sharma and Kanneganti, 2021). In addition, NLRP3 inflammasome can modulate the recruitment and polarization of TAMs and myeloid-derived suppressor cells (MDSCs), which secrete proinflammatory mediators that support tumor progression (Faria et al., 2021). Over-activation of NF-κB, signal transducer and activator of transcription (STAT) signaling, and hypoxia-inducible factor-1α (HIF-1α), a crucial transcription factor, is more significant in bladder cancer by upregulating the expression of NLRP3 and pro-IL-1β and the activation of pyroptosis (Jiang et al., 2020), resulting in an aberrant rise in inflammatory cytokines and immune cell over-response, encouraging cancer (Xu G. et al., 2021). A variety of inflammation-induced diseases, and genetic variation in the NLRP3 inflammasome pathway gene, are linked, for this specific tumor, to the development of malignant and aggressive forms (Song et al., 2012). The NLRP3 inflammasome also plays a crucial role in colorectal cancer (CRC) development, particularly in the advanced stage for his role as a major regulator of intestinal homeostasis and microbiota composition (Vafaei et al., 2022). Other signaling pathways that interact with NLRP3 inflammasome, such as NF-κB, HIF-1α, and STAT3, can be modulated, negatively CRC development (Lin et al., 2020). NLRP3 is required for melanoma growth, progression, and immune response (Wang et al., 2021). According to various studies, NLRP3 expression may influence the B-T cells compositional ratio and macrophages in the immunological microenvironment of Skin Cutaneous Melanoma (SKCM) tumor tissue, therefore indirectly modulating the expression of programmed death-ligand 1 (PD-L1) on melanoma cells, which suppresses the activation and function of CD8^+^ T cells, influencing tumor progression. Additionally, the activation of NLRP3 inflammasome in melanoma cells can promote cell resistance to death by regulating autophagy and mitochondrial energy production (Sharma and Kanneganti, 2021). In addition, NLRP3 expression was significantly positively correlated with infiltrating levels of all immune cell types that influence the TME and regulate tumor behavior (Wang et al., 2022). In fact, Ping Wang et al. found that high infiltration levels of macrophages were significantly associated with reduced survival, probably by regulating polarization of M2 phenotype. Moreover, positive correlations of NLRP3 with Treg and T-cell exhaustion markers indicated that it may induce T-cell exhaustion by activating Tregs. These correlations suggested that NLRP3 is involved in regulating T-cell functions (Wang et al., 2022). A remarkable finding is that NLRP3 had positive correlations with some important immune checkpoint genes, such as TIM-3, PD-1, CTLA4, and LAG3. High expression of these molecules can deplete T cells, thereby reducing immune surveillance, suppressing the killing of tumor cells, and eventually leading to immune escape of tumor cells (Chen F. et al., 2021). Moreover, NLRP3 signaling is influenced by a variety of factors, including genetic polymorphisms and mutations that change gene expression and contribute to its activation (Hamarsheh and Zeiser, 2020). Mutations also affect the function of NLRP3 in specific tumors by affecting tumor type, tumor stage, and effector molecules downstream of NLRP3 signaling. As a result, NLRP3 pathway has complicated consequences on tumor initiation and development. Therefore, understanding the mechanisms and consequences of NLRP3 inflammasome activation in cancer is critical for developing novel therapeutic strategies (Hamarsheh and Zeiser, 2020) (Figure 2).

## 4 Unravelling the significance of NLRP3 in oral cancer

**FIGURE 3:**
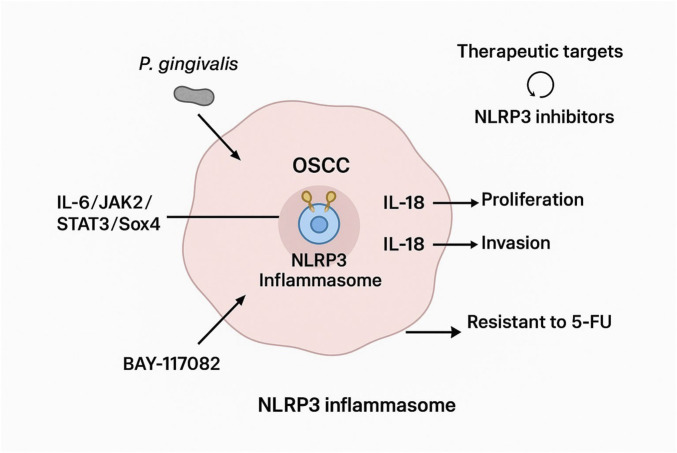
Mechanistic significance of NLRP3 inflammasome activation in OSCC. NLRP3 activation OSCC is regulated by multiple upstream stimuli, including chronic microbial exposure (e.g., Porphyromonas gingivalis) and inflammatory cytokine signaling via the IL-6/JAK2/STAT3/SOX4 pathway. Once assembled, the NLRP3 inflammasome drives caspase-1 activation and the maturation of IL-1β and IL-18, which in turn promote tumor-cell proliferation, invasion, epithelial–mesenchymal transition (EMT), angiogenesis, and remodeling of the tumor microenvironment. NLRP3 signaling also contributes to immune evasion and enhances resistance to 5-fluorouracil (5-FU), supporting a more aggressive OSCC phenotype. Pharmacologic inhibition of NLRP3, including agents such as BAY-117082 or next-generation NLRP3 inhibitors, has demonstrated the ability to reduce inflammasome activity and suppress OSCC progression, highlighting this pathway as a promising therapeutic target.

Recent evidence has increasingly implicated the NLRP3 inflammasome in the pathogenesis of OSCC, highlighting its dual role in promoting both chronic inflammation and tumor progression. OSCC is usually correlated with multifaceted process of carcinogenesis and poor prognosis. These particularities emphasize the crucial need for improved detection, earlier diagnosis, effective treatments, and better patients oriented prognostic decisions (Jain et al., 2024). In this context, since the expression of NLRP3 protein and other factors of this pathway is not well characterized in OSCC and Potentially Malignant Oral Disorders (PMODs), a lot of studies aim to explore NLRP3 inflammasome/IL-1β signaling pathway composed with the inflammatory microenvironment and tumor immunity, their role in OSCC and their potentially association with pathological parameters including tumor size, depth of invasion (DOI), pTNM stage, and perineural and lympho vascular invasion. Overexpression of NLRP3, along with elevated levels of its downstream effectors such as IL-1β and IL-18, has been consistently observed in OSCC tissues compared to adjacent normal mucosa (Wang et al., 2018). Cytokines in the TME are produced by a variety of cell types and play their autocrine and paracrine roles. Recently a lot of studies aim to explore the role of IL-1β in inflammatory microenvironment and tumor immunity and the potentially correlation with this molecule and different clinical-pathological parameters in OSCC. IL-1β is commonly recognized as a major tumor-promoting cytokine and his role is associated with unfavourable prognostic outcomes linked with cancer-related inflammation and compromised immune function (Huang C. F. et al., 2017; Chen L. et al., 2018). Moreover, there is a significant correlation between CAFs and TAMs, usually the most abundant cells in oral cancer TME, the release and maturation of IL-1β. TAMs secrete cytokines, chemokines, and enzymes that stimulate cell growth, differentiation, tumor progression and angiogenesis (Kaneko et al., 2019). Furthermore, CAFs are continuously activated in TME and can promote tumorigenic features by initiating remodeling of the extracellular matrix (ECM) and secreting cytokines. As well, metabolites, secreted by same cells, are an important component in TME, providing energy for tumor progression (Chen X. et al., 2021). These evidence, indicated that there is a molecular cross talk between cancer cells and surrounding stroma playing an important role for enhancing tumor growth and progression (Wang et al., 2019). Thus, targeting CAFs or TAMs is important for understanding cancer invasion and finding keys to suppress OSCC cancer progression (Zhang and Hwang, 2019). NLRP3 inflammasome/IL-1β signaling pathway have been associated with advanced pathological stages, deeper invasion, nodal metastases, and reduced overall survival, highlighting their potential as prognostic biomarkers. Firstly, the maturation and release of IL-1β and IL-18 mediated by activation of caspase-1 can enhance the chronic inflammatory response in OSCC through the induction of DNA damage and the malignant transformation of epithelial cells (Liston and Masters, 2017). Moreover, the secretion of IL-1β can recruit immunosuppressive cells, such as regulatory T cells and M2 macrophages (Xue et al., 2019), that can inhibit the anti-tumor immune response, and promote angiogenesis and matrix remodeling, providing tumor progression. In addition, OSCC is characterized by hypoxia and ROS accumulation, or other common metabolic abnormalities that may further exacerbate inflammation and tumor invasion by the activation of NLRP3 (Figure 3). Also pyroptosis plays a dual role in OSCC (Mangan et al., 2018). On the one hand, the cellular contents released during pyroptosis promote local inflammation, supporting tumor metastasis, but on the other hand, excessive pyroptosis may also limit the proliferation of tumor cells. Thus, the specific effects depend on the tumor stage and other signals in the TME (Sh et al., 2025). OSCC often arises from PMODs such as leukoplakia, erythroplakia, oral lichen planus, and oral submucous fibrosis, highlighting the importance of early detection and intervention. Functional studies have confirmed that genetic knockdown of NLRP3 significantly impairs OSCC cell proliferation, migration, and invasiveness *in vitro*, while also reducing tumorigenic potential in xenograft models (Wang et al., 2018).

Furthermore, emerging data link NLRP3 activation to key oncogenic signaling pathways. For instance, IL-6 has been shown to activate NLRP3 via the JAK2/STAT3/SOX4 axis, effectively connecting inflammatory cytokine signaling with inflammasome-mediated tumor enhancement (Zhou et al., 2023). This axis promotes EMT, stemness, and immune evasion, traits strongly associated with aggressive tumor phenotypes. Additionally, recent findings suggest that NLRP3 may contribute to a pro-tumorigenic microenvironment by modulating macrophage polarization and promoting the recruitment of MDSCs, although this aspect remains under investigation.

**FIGURE 4:**
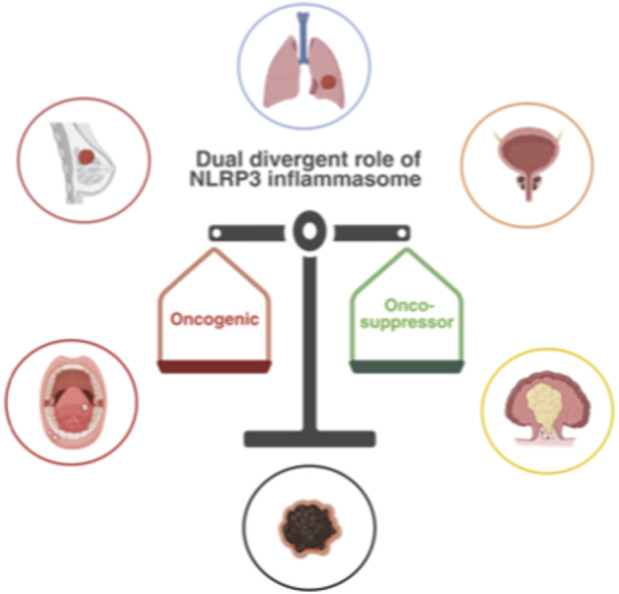
MicroRNAs targeting NLRP3 pathway as a prognostic and diagnostic biomarker. **(A)** Higher expression of the NLRP3 factor is commonly correlated with different clinicopathological parameters in OSCC and various regulation of molecular pathways through immune- and tumor-related signaling. Therefore, it is regarded as a promising prognostic and diagnostic marker in OSCC. **(B)** Next, there is represented the possibility of targeting NLRP3 complex using different types of microRNAs, which could induce repression of NLRP3 mRNA by miRNA RISC complex and NLRP3 mRNA degradation as an innovative outcome to downregulation of NLRP3 inflammosome signaling (created by BioRender).

Thus, NLRP3 may be a potential risk factor for invasion and metastasis, effectively contributing to poor prognosis. Consequently, NLRP3 is considered a promising target for prognostic assessment, diagnostics, and therapeutic strategies in OSCC. Taken together, these findings emphasize the central role of NLRP3 in driving both intrinsic tumor properties and extrinsic inflammatory cues in OSCC, offering a strong rationale for targeting this inflammasome in future therapeutic strategies (Figure 4).

## 5 microRNA as important regulators of NLRP3 inflammosome

**FIGURE 5:**
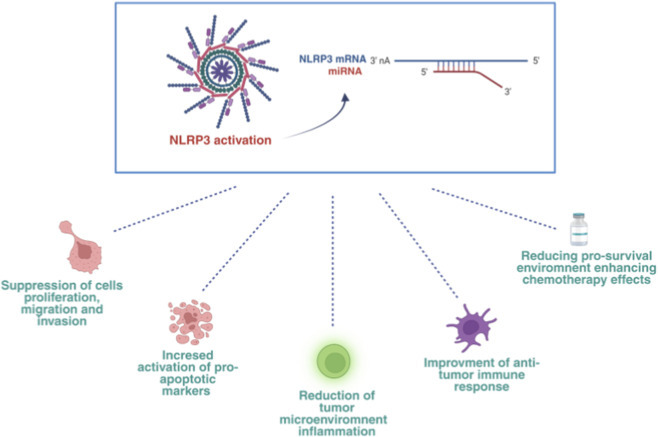
NLRP3 activation and miRNA regulation in OSCC pathogenesis. The modulation of NLRP3 via miRNA that regulates the level of inflammasome complex expression, also modulates a plethora of downstream key effects in tumor progression, invasiveness, and response to therapy. A downregulation of NIRP3 reduces IL-1β secretion and associated tumor inflammation. The regulated pathway restores the expression of pro-apoptotic markers and has an important role in the enhancement of chemotherapy effectiveness (created with BioRender).

Since 2012, NLRP3 has emerged as a direct target of post-transcriptional regulation by microRNAs (miRNAs), adding an additional layer of control to the complex mechanisms governing its expression and inflammasome activation (Lin et al., 2025; Boxberger et al., 2019). That year, miR-223-3p was identified as the first human miRNA directly targeting the 3′untranslated region (3′UTR) of NLRP3 mRNA, revealing an additional layer of inflammasome regulation and underscoring the importance of miRNAs in innate immunity (Haneklaus et al., 2012; Bauernfeind et al., 2012). miRNAs are small non-coding RNAs (∼20–25 nucleotides) that repress gene expression by binding to complementary sites in target mRNAs, leading to mRNA degradation or translational inhibition via the RNA-induced silencing complex (RISC) (Kim and Croce, 2023). Their biogenesis involves sequential processing by Drosha and Dicer enzymes, resulting in mature miRNAs that guide RISC to specific mRNA targets (Miyoshi et al., 2010). Through this regulatory mechanism, miRNAs orchestrate crucial cellular processes such as proliferation, differentiation, apoptosis, and metabolism, thereby maintaining cellular homeostasis (Kim and Croce, 2023). Since the discovery of the first miRNA (lin-4) in *Caenorhabditis elegans*, thousands of miRNAs have been identified across species. Currently, the human genome is believed to encode more than 2500 miRNAs and their dysregulation has been implicated in a wide range of human diseases, including cancer (Ambros, 2004; Lee et al., 1993). In oncology, miRNAs can act as either oncogenes or tumor suppressors depending on the cellular context and each cancer type features unique miRNA expression patterns (Kim et al., 2011) (Figure 5). The regulatory interaction between miR-223-3p and NLRP3 was originally described in the context of monocyte-to-macrophage differentiation, during which miR-223-3p expression decreases while NLRP3 levels rise (Haneklaus et al., 2012). A cell-type–specific gradient has also been observed: miR-223-3p is most abundant in neutrophils and lowest in dendritic cells, suggesting a role in modulating inflammasome sensitivity (Bauernfeind et al., 2012). Furthermore, mutations in the NLRP3 3′UTR can abolish miR-223-3p binding, highlighting the specificity and importance of this regulatory axis (Neudecker et al., 2017).

Functionally, miR-223-3p acts as a negative regulator of NLRP3 across multiple pathological settings. In cancer models, such as breast cancer, miR-223-3p inhibits NLRP3 activation, suppresses tumor progression, and enhances anti-tumor immune responses (Zhang et al., 2019). It also plays a role in cartilage degeneration (Dong et al., 2019), immune cell regulation (Pachathundikandi and Backert, 2018), and has been linked to pyroptosis in colitis (Wu et al., 2020). In reflux esophagitis, miR-223-3p appears to limit inflammation and epithelial damage by targeting NLRP3, although its expression decreases from the acute to the chronic phase, suggesting a dynamic and stage-dependent role (Uemura et al., 2017). Interestingly, viral miRNAs may exploit this regulatory pathway. The Epstein-Barr virus (EBV)–derived EBV-miR-BART15-3p targets the same site on NLRP3 mRNA as miR-223-3p and can be transferred to non-infected cells via exosomes to suppress inflammasome activation, likely a viral immune evasion strategy. A polymorphism (rs10802501) in the binding site may modulate the interaction of both miRNAs with NLRP3 (Haneklaus et al., 2012). In addition to miR-223-3p, other miRNAs have been shown to regulate NLRP3 inflammasome activity. For instance, miR-7-5p and miR-30e-5p suppress inflammasome priming and alleviate Parkinson’s disease-like phenotypes in α-synuclein-based mouse models (Fan et al., 2016; Li et al., 2018; Zhou et al., 2016), while miR-20b-5p reduces inflammation in *Mycobacterium tuberculosis*-infected lung tissue (Lou et al., 2017). miR-22-3p exerts protective effects in coronary artery disease by downregulating NLRP3 in injured endothelial cells (Huang W. Q. et al., 2017) and suppresses cell proliferation, migration, and invasion in OSCC by targeting NLRP3 (Feng et al., 2018). miR-133b-3p modulates allergic rhinitis by dampening NLRP3-mediated inflammation (Xiao et al., 2017). Moreover, miR-186-5p and miR-495-3p demonstrate anti-inflammatory roles in models of neuropathic pain and myocardial ischemia/reperfusion injury, respectively, via targeting of NLRP3 (Chen M. L. et al., 2018; Zhou et al., 2018) (Table 1).

## 6 Therapeutic implications

Given the involvement of the NLRP3 inflammasome in numerous inflammatory and tumor conditions, the development of molecules capable of modulating its activity has become a central goal of pharmacological research. However, despite significant progress, the clinical application of NLRP3 inhibitors remains complex and still far from full translation into oncology. Early NLRP3 inhibitors included indirect modulators such as resveratrol, arglabine, glyburide, and β-hydroxybutyrate (BHB). However, these compounds affected multiple signaling pathways and caused significant off-target effects. To overcome these limitations, several selective NLRP3 inhibitors have been developed, including MCC950, BAY-117082, CY-09, OLT1177, tranilast, MNS, and oridonin. These molecules target NLRP3 or its oligomerization more specifically and therefore reduce off-target immunosuppression. By directly targeting the NLRP3 protein or its complex, these inhibitors can reduce unwanted side effects and off-target immunosuppressive effects (Coll et al., 2015). Over the past decade, studies have focused on investigating there *in vitro* and *in vivo* effects on inflammatory diseases, neurodegenerative diseases, and some types of cancer (Casili et al., 2024). Among these inhibitors, the small molecule NLRP3 inhibitor MCC950 has shown promising results in various disease models, including autoinflammatory disorders, cardiovascular diseases, cancer, neurological diseases, and diabetes. MCC950 inhibits ATP hydrolysis and inflammasome formation by binding to the NACHT domain of the NLRP3 protein (Coll et al., 2019). Another inhibitor, BHB, reduces K^+^ efflux and ASC aggregation, thereby preventing NLRP3 activation and lowering IL-1β and IL-18 levels in human monocytes. BHB has shown anti-inflammatory effects in models of Alzheimer’s disease, gout, and acute kidney injury and has potential as a clinical treatment (Shippy et al., 2020). However, the clinical use of NLRP3 inhibitors still faces challenges related to pharmacokinetics and hepatotoxicity (Figure 1). Despite these advances, the translational development of NLRP3 inhibitors remains limited. Their clinical applicability is currently limited by suboptimal pharmacokinetic profiles, the risk of systemic immunosuppression, and hepatotoxicity, factors that limit their long-term use in oncology (Nizami et al., 2021). Furthermore, although new-generation inhibitors demonstrate greater specificity for the NLRP3 complex, they still lack tumor-selective activity, raising concerns about off-target effects in tissues with physiological inflammasome functions (Zahid et al., 2019). Importantly, only a few NLRP3-targeting compounds, such as DFV890, have entered early-stage clinical trials, and none have yet progressed to cancer studies (Zahid et al., 2019). Therefore, the therapeutic potential of these agents in oncology remains largely unexplored in clinical settings.

In light of these limitations, increasing attention has shifted toward alternative strategies capable of modulating NLRP3 activity with potentially greater precision and fewer systemic effects. Among these, microRNA-based therapeutic approaches represent a particularly innovative avenue, offering the possibility of indirectly regulating the inflammasome upstream of its activation. Preclinical data indicate that miR-223 downregulates NLRP3 and attenuates inflammasome activation, whereas miR-155 enhances NLRP3-driven inflammatory signaling (Xia et al., 2021). Although still at an early stage, the antisense inhibitor of miR-155 cobomarsen (MRG-106) has demonstrated safety and target engagement in phase I clinical trials for lymphoid malignancies. While not developed specifically to modulate NLRP3, its ability to suppress a key upstream activator of the inflammasome provides an indirect yet clinically relevant mechanism of NLRP3 regulation. Overall, these findings suggest that microRNA-directed therapies may offer a more refined and clinically feasible approach to controlling pathological NLRP3 activation, potentially overcoming some of the translational limitations associated with direct NLRP3 inhibitors (Table 2).

**TABLE 2:** Summary table of pharmacological inhibitors of NLRP3 inflammasome and their effects in molecular mechanisms in different types of cancer.

Cancer types	Inhibitors	Outcomes	References
OSCC	BAY-117082	• Inhibition of key factors of inflammasome complex • Reduction of pro-inflammatory cytokines • Block of NF-kB translocation	Casili et al. (2023) Scuderi et al. (2021)
Breast cancer OSCC Pancreatic cancer Head and neck squamous cell carcinoma	MCC950	• Reduction of tumor-related inflammation and IL-1β production • Reduction of tumor cells proliferation • Improvement of anti-tumor responses	Yaw et al. (2020) Balahura Stamat and Dinescu (2024) Hamarsheh and Zeiser (2020)
Osteosarcoma	CY-09	• inhibition the NLRP3 inflammasome by directly binding NLRP3 protein blocking ATPase activity of NACHT domain • Block oligomerization and assembly of NLRP3 complex and subsequently its activation	Huang Z. et al. (2021)
Melanoma	OLT1177	• Reduction of NLRP3-ASC and NLRP3-caspase-1 interaction • Reduction of ATPase activity • Decreased production of IL-1β and IL-18	Tengesdal et al. (2021)
Bladder cancer Breast cancer Liver (hepatocellular carcinoma)	Tranilast	• Inhibition of NLRP3 oligomerization • Block NLRP3-ASC complex and caspase-1 activation • Reduction of IL-1β production	Huang et al. (2018), Chen et al. (2020)
Breast cancer	MNS	• Inhibition of NLRP3 complex targeting the nucleotide binding oligomerization domain (NACHT) and leucine-rich repeat (LRR) domains • Suppression of downstream release of pro-inflammatory cytokines like IL−1β and IL−18	Selvanesan et al. (2023)
Breast cancer Hepatocellular carcinoma Colorectal cancer Leukemia Lymphoma Gastric cancer Lung cancer Pancreatic cancer	Oridonin	• Covalent inhibition of NACHT domain and block of interaction between NLRP3 and NEK7 • Suppression ASC oligomerization and caspase-1 activation • Downregulation of IL−1β production	Selvanesan et al. (2023) Yang et al. (2020) He et al. (2018)

## 7 Future perspective

Given its multifaceted role in OSCC progression, targeting NLRP3 represents a promising and innovative therapeutic approach. Pharmacological inhibition using small-molecule compounds such as BAY-117082 has shown substantial efficacy in preclinical models by downregulating key inflammasome components (NLRP3, ASC, caspase-1) and reducing the production of pro-inflammatory cytokines IL-1β and IL-18. This was accompanied by increased activation of pro-apoptotic markers such as p53, Bax, and Bad, ultimately leading to decreased tumor burden *in vivo* (Scuderi et al., 2021). These results suggest that NLRP3 inhibition may not only halt tumor growth but also enhance tumor cell susceptibility to apoptosis. Moreover, the inflammasome has been implicated in mediating chemoresistance in OSCC. Activation of the ROS/NLRP3/IL-1β axis in response to 5-FU treatment appears to contribute to a pro-survival environment, limiting the efficacy of standard chemotherapy. Inhibition of NLRP3 in this context has been shown to restore chemosensitivity and increase apoptotic responses in tumor cells (Feng et al., 2017).

However, from a translational perspective, several critical challenges must be addressed before NLRP3-targeted strategies can enter clinical evaluation. Currently available NLRP3 inhibitors lack cancer specificity, and their systemic immunosuppressive effects raise concerns (Schwaid and Spencer, 2021). The development of next-generation tumor-selective inhibitors or locally deliverable formulations (e.g., topical, intratumoral, or nanoparticle-based systems) will be essential to minimize off-target toxicity, particularly in the oral cavity, where mucosal integrity is crucial. Furthermore, patient stratification remains an unresolved issue, as the heterogeneity of OSCC means that only a subset of patients may benefit from NLRP3 blockade. Future studies should clarify whether NLRP3 expression levels or specific miRNA profiles can serve as reliable biomarkers to identify patients who will respond to treatment. Non-coding RNAs, particularly miRNAs, have recently emerged as key modulators of NLRP3 activity. miRNAs such as miR-223-3p and miR-22-3p have demonstrated the ability to suppress NLRP3 expression and downstream signaling, suggesting a potential role therapeutic agents or adjuvants. However, the clinical translation of miRNA-based therapies poses additional challenges, including stability in biological fluids, efficient targeted delivery, and the prevention of unwanted immune activation. Advanced delivery strategies, such as lipid nanoparticles, exosome-mimicking vesicles, or biomaterial-based scaffolds, should be explored to improve specificity and therapeutic index. Another major opportunity lies in the integration of NLRP3 inhibition into combination treatment paradigms. Because the inflammasome orchestrates crosstalk between tumor cells and the immune microenvironment, its blockade could potentiate immunotherapeutic approaches such as PD-1/PD-L1 inhibitors or modulate tumor-associated macrophage polarization. However, this synergy has yet to be validated in OSCC-specific models, and careful evaluation of immune-related adverse events will be required. Similarly, incorporating NLRP3 inhibitors into chemoradiation regimens may enhance tumor sensitivity but may also interfere with inflammation-dependent tissue healing, necessitating precise timing and dosing strategies. Ultimately, a deeper understanding of the interplay between NLRP3, the tumor microenvironment, and immune regulation will be critical for designing effective therapeutic strategies. Well-designed preclinical models, biomarker-driven clinical trial frameworks, and optimized delivery platforms will collectively determine whether NLRP3-targeted interventions can transition from experimental concept to viable clinical therapy for OSCC.
